# Unlocking the potential of biocrust microorganisms in agriculture: cyanobacteria and heterotrophic bacteria with plant growth-promoting properties

**DOI:** 10.3389/fpls.2025.1659217

**Published:** 2025-09-24

**Authors:** Carlotta Pagli, Lisa Maggioli, Beatriz Roncero-Ramos, Eloisa Pajuelo, Miriam Muñoz-Rojas, Roberto Braglia, Antonella Canini, Yolanda Cantón

**Affiliations:** ^1^ Department of Agronomy, University of Almería, Almería, Spain; ^2^ Department of Biology, University of Rome Tor Vergata, Rome, Italy; ^3^ Departamento de Biología Vegetal y Ecología, Facultad de Biología, Universidad de Sevilla, Sevilla, Spain; ^4^ Department of Microbiology and Parasitology, Faculty of Pharmacy, University of Seville, Seville, Spain; ^5^ Laboratorio de Biodiversidad y Funcionamiento Ecosistémico, Instituto de Recursos Naturales y Agrobiología de Sevilla (IRNAS), Consejo Superior de Investigaciones Científicas (CSIC), Sevilla, Spain

**Keywords:** plant growth promotion (PGP), biocrust, cyanobacteria, heterotrophic bacteria, sustainable agriculture

## Abstract

**Introduction:**

Drylands are subject to multiple overlapping stresses, including high temperatures, drought, and salinity, along with soils that are low in organic matter and nitrogen. Hence, both agricultural practices and natural regeneration in these areas are hindered by poor plant establishment and growth. The use of plant growth-promoting (PGP) microorganisms has recently emerged as a promising strategy to enhance plant performance under these harsh conditions.

**Methods:**

In this context, the aim of this work was to isolate and screen the PGP properties of cyanobacteria and heterotrophic bacteria from biocrusts in arid soils, representing a highly unexplored niche of microorganisms with potential application in agriculture and ecological restoration. We determined key PGP traits, including phosphate and potassium solubilization, growth under nitrogen-free conditions, siderophore and auxin production, as well as protease, lipase, DNase, amylase, catalase, and cytochrome-C-oxidase activities.

**Results:**

Our results showed that, among the cyanobacteria analyzed, *Nostoc commune* CANT2, isolated from the province of Almería (Spain), exhibited the highest number of PGP properties, followed by *N. commune* AB55 (southern Sardinia, Italy) and *Trichocoleus* cf. *desertorum* CAU7 (Almería). Both strains AB55 and CANT2 are characterized by their production of exopolysaccharides (EPS). Regarding the heterotrophic bacterial strains, those with the best PGP properties were identified as *Peribacillus frigoritolerans* and *Bacillus atrophaeus* by 16S rRNA gene sequencing. Seed biopriming experiments with the model plant *Triticum aestivum* showed that application of *N. commune* CANT2, either alone or in combination with *P. frigoritolerans* 1E, enhanced vigor indices by up to 58% compared to the control.

**Discussion:**

These findings highlight the potential of combined microbial consortia with PGP activities as candidates for the development of biostimulants, offering a sustainable approach to improve plant growth and resilience in dryland agriculture.

## 1 Introduction

At a global level, cultivated land collectively accounts for 38% of the Earth’s surface, with a portion of soil suitable for cultivation up to 4.4 billion hectares (Zabel et al., 2019; Hu et al., 2020). Because of population growth, economic development, and increasing food demand, this percentage is expected to increase (Wang et al., 2023). Over the past 40 years, 30% of arable lands have become unproductive because of high erosion rates, pollution, and nutrient loss (Giller et al., 2021), risking the production of food for global population. Thus, the need to preserve soil functions has been included in the Sustainable Development Goals (SDGs) of the 2030 agenda, approved by the United Nations General Assembly in 2015 (UN General Assembly, 2015). Agricultural soil degradation primarily stems from the combined effects of climate change and the intensification of agricultural practices (Pereira et al., 2023). Some soil-degrading processes are closely related to agriculture, such as water, wind, and mechanical erosion due to soil tillage, compaction, reduction of organic carbon content, biodiversity loss, salinization, as well as soil contamination by heavy metals, pesticides, and excessive chemical fertilizers (Lehmann et al., 2020; Prăvălie et al., 2021; Gupta et al., 2022). In particular, the excessive and indiscriminate use of chemicals in agriculture has led to food contamination, the development of resistance to them by weeds and pathogens, as well as deleterious environmental effects (Bajwa and Sandhu, 2014; Okon and Antia, 2023). Therefore, finding innovative solutions and approaches to support the development of sustainable, environmentally friendly agriculture has become a priority.

The use of plant growth-promoting microorganisms (PGPM) has emerged as a promising solution to enhance sustainable agricultural practices, simultaneously offering an alternative to chemical fertilizers, pesticides, and amendments (Mishra et al., 2017; Naik et al., 2019). PGPM constitute a group of beneficial microorganisms, including both heterotrophic and photoautotrophic bacteria, fungi, and archaea, which can establish symbiotic relationships with plants. These microorganisms play a key role facilitating nutrients assimilation, improving soil structure, and mitigating environmental stress factors (Chukwuneme et al., 2020; Flores-Duarte et al., 2022). In this context, heterotrophic bacteria and cyanobacteria stand out as promising candidates due to their unique plant growth-promoting (PGP) properties. Different studies have investigated the capacity to fix atmospheric nitrogen, an essential nutrient for plants, exhibited by these microorganisms, thereby promoting soil fertility (Soumare et al., 2020; Ambrosio et al., 2022; Khumairah et al., 2022; Rana et al., 2023). Additionally, several investigations have revealed the successful application of isolated PGPM in phosphorus and potassium solubilization, enhancing nutrient uptake and promoting plant growth (Silva et al., 2023; Bispo et al., 2023; Gandhi et al., 2023). The capacity of both heterotrophic bacteria and cyanobacteria to produce siderophores, which chelate iron and other essential nutrients, further underscores their crucial role in nutrient mobilization and availability for plants (Chakraborty et al., 2019; Garg et al., 2021; Timofeeva et al., 2022). Siderophores not only enhance the bioavailability of iron but also aid in the uptake of other nutrients, such as phosphorus, by forming soluble nutrient complexes (Cui et al., 2022). This process promotes a more favorable rhizospheric environment for optimal plant growth (Ghosh et al., 2020; Timofeeva et al., 2022). Moreover, these molecules have function in biocontrol by competing for Fe with phytopathogens (Di Francesco and Baraldi, 2021). Some heterotrophic bacteria and cyanobacteria strains can also synthesize and secrete phytohormones, such as auxins, playing a crucial role in various plant processes, including cell elongation, root development, and overall growth (Nazli et al., 2020; Duong et al., 2021; Orozco-Mosqueda et al., 2023). Some microorganisms, including heterotrophic bacteria and cyanobacteria, can produce biofilms (Tilahun et al., 2016; Potnis et al., 2021) which act as protective microenvironments for microbial communities, enhancing their resilience against environmental stressors and promoting nutrient exchange (Mahto et al., 2022). The ability of microorganisms to form biofilms can significantly influence their effectiveness as biostimulants, as these structures enhance their stability and persistence in the soil, fostering beneficial interactions with plants in the rhizosphere (Pandit et al., 2020; Parrilli et al., 2022; Brokate et al., 2024). In addition, various enzymatic activities—such as lipase, protease, DNase, and amylase—are usually evaluated to assess the effectiveness of heterotrophic bacteria and cyanobacteria as plant growth promoters. These hydrolytic enzymes contribute to the breakdown of organic matter and the release of essential nutrients, thereby enhancing soil fertility and improving plant nutrition (Omoarelojie et al., 2021; Saeed et al., 2021; Neemisha and Sharma, 2022; Liu et al., 2023; Daunoras et al., 2024). In addition, some of these enzymes, such as cellulases, pectinases and xylanases, are involved in facilitating the colonization of plant tissues by beneficial microorganisms acting as endophytes (Dogan and Taskin, 2021). These enzymatic activities reflect the microorganisms’ ability to actively participate in nutrient cycling and promote plant health, which is especially relevant in agricultural applications.

In recent years, PGP properties have been extensively studied, particularly in heterotrophic bacteria and, to a lesser extent, in cyanobacteria (Gupta et al., 2018; Ferreira et al., 2019; Múnera-Porras et al., 2020). Moreover, recent research has focused on isolating PGPM primarily from agricultural soils, with a specific emphasis on the rhizosphere of crop plants (Gu et al., 2020; Nazli et al., 2020; Zhao et al., 2020; Khumairah et al., 2022). Therefore, the isolation of heterotrophic bacterial and cyanobacterial strains from new niches is crucial to expand the collections of microorganisms accessible to be used in biofertilization, phytostimulation, and for biocontrol purposes.

One promising and unexplored source of PGPM are biological soil crusts, also known as biocrusts. Despite their potential, PGPM from biocrusts remain largely unexplored. Only a few studies have investigated their diversity and plant growth-promoting abilities, leaving a vast microbial resource untapped. This gap highlights the novelty and relevance of exploring biocrust-derived microorganisms for sustainable agriculture applications. Biocrusts are complex communities that can include cyanobacteria, heterotrophic bacteria, lichens, and mosses, among other organisms, that form a protective layer within the first centimeter of soil surfaces (Weber et al., 2022). In these communities, cyanobacteria (photoautotrophs) and heterotrophic bacteria are intimately associated, and their interactions are crucial for sustaining balanced soil ecosystem functioning (Moreira-Grez et al., 2019). Cyanobacteria fix carbon and nitrogen, providing essential substrates for heterotrophs, which in turn enhance nutrient availability, soil structure, and water retention (Delgado-Baquerizo et al., 2013; Dadzie et al., 2024). Their close physical proximity facilitates metabolite exchange, accelerating biocrust establishment and amplifying the benefits of each group compared to their isolated effects (Nelson and Garcia-Pichel, 2021; Zheng et al., 2023). This synergy not only supports soil stabilization and nutrient cycling but also offers promising applications in sustainable agriculture (Dadzie et al., 2022). The ability of bacteria and cyanobacteria from biocrusts to survive in harsh environments, such as arid and semi-arid regions, highlights not only their resilience but also their potential for use in sustainable agriculture (Chen et al., 2020; Bashtian et al., 2024). In the context of climate change, with expected decreases in precipitation and increased soil salinization, their capacity to withstand stresses like desiccation and high salinity could provide a significant advantage, enabling them to thrive under challenging field conditions (Fan et al., 2023; Roque et al., 2023). Indeed, these microorganisms can help alleviate stressful conditions for plants by providing essential nutrients, immobilizing salts, and improving overall soil quality, which ultimately enhances the plant’s ability to withstand various stressors (Kumar and Verma, 2018; Koza et al., 2022; Hnini et al., 2024). Also, biocrust cyanobacteria have shown biocontrol potential against soilborne pathogenic fungi (Águila-Carricondo et al., 2024). The distinctive diversity of biocrust microorganisms combined with their ability to produce a variety of bioactive substances, including enzymes and phytohormones, which can positively influence plant growth and development, make biocrusts a valuable source to obtain new strains of microorganisms with key PGP properties (Steven et al., 2014; Li et al., 2021).

Hence, this research aims to improve agricultural practices by obtaining heterotrophic bacterial and cyanobacterial strains from biocrusts with beneficial PGP properties. The main goals of this work were to (i) isolate native heterotrophic bacteria strains from biocrusts collected from semi-arid study sites, (ii) characterize the functional traits associated with plant growth promotion and enzymatic activities of both newly isolated heterotrophic bacteria and previously identified biocrust-forming cyanobacteria, and (iii) test the effect of seed biopriming with a consortium of the best performing PGP cyanobacteria and bacteria on the germination and initial development of a crop model plant (*Triticum aestivum*).

## 2 Materials and methods

### 2.1 Isolation of heterotrophic bacteria from biocrust

**Figure 1:**
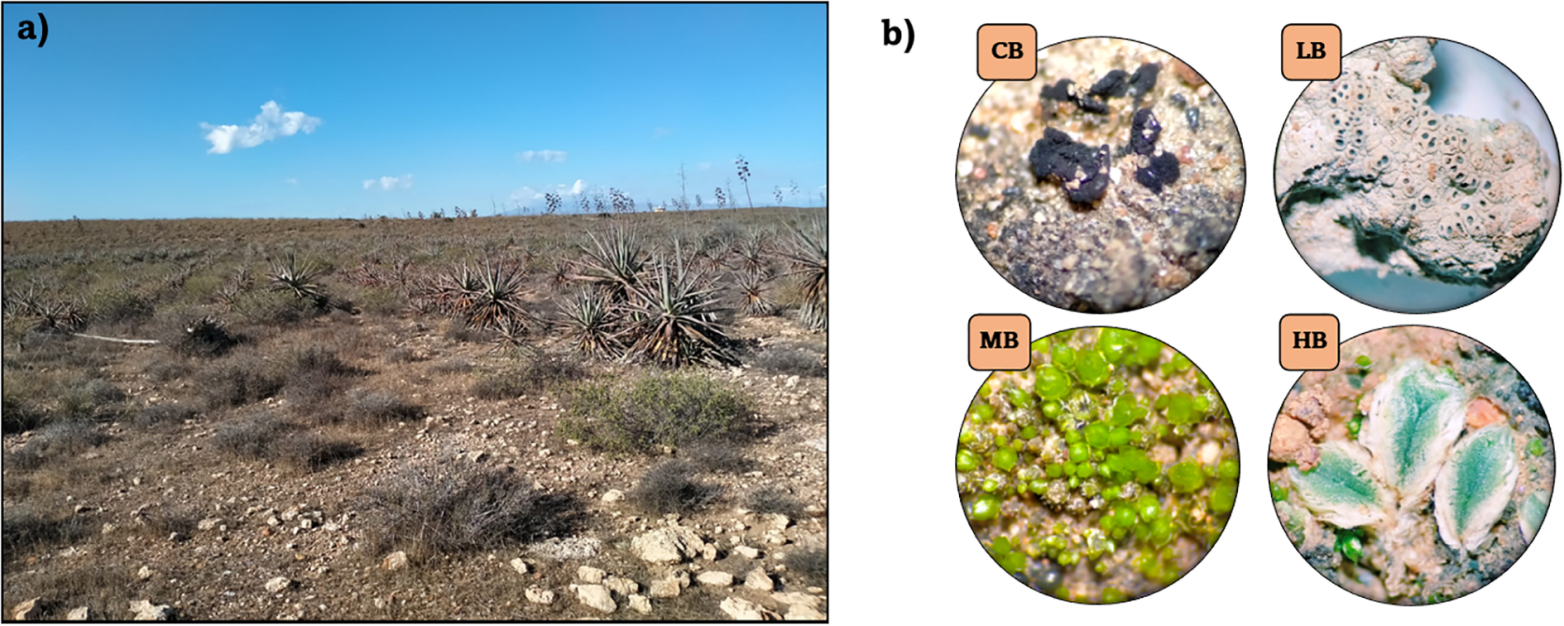
**(a)** Overview of the sampling site: Las Amoladeras, Almería. **(b)** Biocrust samples: cyanobacteria-dominated biocrust (CB), lichen-dominated biocrust with Diploschistes sp. as the dominant species (LB), moss-dominated biocrust (MB), Hepatophyta-dominated biocrust (HB). Observed under a stereomicroscope at 4.5x magnification.

Biocrust samples were collected in December 2020 from Las Amoladeras experimental site (N 36° 50′ 01″ W 02° 15′ 08″), which is part of the Mediterranean grassland ecosystem within the Cabo de Gata‐Níjar Natural Park (Almería, SW Spain) (**Figure 1**). The site is characterized by a semi-arid Mediterranean climate, with mean annual rainfall of 200 mm, mean annual temperature of 18°C, and distinctive vegetation adapted to drought conditions (Chamizo et al., 2012; Román et al., 2018). Different types of biocrusts at various succession stages were collected: cyanobacteria-dominated biocrust (CB), lichen-dominated biocrust with *Diploschistes* sp. as the dominant genus (LB), moss-dominated biocrust (MB), and Hepatophyta-dominated biocrust (HB).

For each biocrust type, three undisturbed samples were collected using a Petri dish and transported to the lab, where they were stored at 4°C for bacterial isolation. Biocrust samples (1 g) were homogenized by mixing with 9 mL of sterile saline solution (0.9% NaCl) in a Falcon tube to achieve a 1:10 dilution. The mixture was thoroughly vortexed and then incubated at 80°C for 30 minutes using a Thermoblock. Serial dilutions of the suspensions were prepared in Eppendorf tubes until reaching a 10–^6^ dilution. Subsequently, 100 µL of each dilution (10^-2^, 10^-4^, and 10^-6^) were inoculated onto Petri dishes containing LB medium at various concentrations (1×, 0.1×, and 0.01×) in triplicate.

This approach, using a non-selective and nutritionally rich medium, facilitated the recovery of diverse heterotrophic bacteria from biocrusts, including both fast- and slow-growing taxa, and supported our exploratory goal of isolating a wide range of cultivable microorganisms potentially involved in PGP activity (Molina-Menor et al., 2021). Petri dishes were then incubated at 23°C in darkness for 48 hours. Different bacteria were separated into different LB plates based on the color and morphology of colonies. Then, pure cultures of each strain were carefully preserved in cryovials containing 20% (v/v) glycerol solution and stored at a freezing temperature of -80°C.

### 2.2 Selection of cyanobacterial strains

The cyanobacteria strains used in this study belong to a collection of microorganisms that were isolated from biocrusts in semi-arid ecosystems located in the province of Almería (Spain) and identified by a morphological and genetic (16S rRNA gene) evaluation (Roncero-Ramos et al., 2019), as well as in the provinces of Medio Campidano and Carbonia-Iglesias in Southwest Sardinia (Italy) (Pagli et al., 2024). The identification and origin of the cyanobacteria strains used in this study are detailed in **Table 1**.

**Table 1:** Species and origins of cyanobacterial strains.

Strain	Species	Origin	Morphotype
AB55	*Nostoc commune*	Ex-mine S. Acqua Bona (SW Sardinia, Italy)	Filamentous, with heterocysts and mucilaginous colonies
NR64	*Nostoc commune*	Naracauli (SW Sardinia, Italy)	Filamentous, with heterocysts and mucilaginous colonies
RI23	*Nostoc commune*	Rio Irvi bank (SW Sardinia, Italy)	Filamentous, with heterocysts and mucilaginous colonies
CANT2	*Nostoc commune*	Gádor quarry (Almería, Spain)	Filamentous, with heterocysts and mucilaginous colonies
CAU7	*Trichocoleus* cf. *desertorum*	El Cautivo (Almería, Spain)	Filamentous, non-heterocystous, branching
CAU10	*Stenomitos frigidus* (previously identified as *Leptolyngbya frigida*)	El Cautivo (Almería, Spain)	Filamentous, non-heterocystous, unbranched
CAU6	*Scytonema hyalinum*	El Cautivo (Almería, Spain)	Filamentous, with heterocysts and false branching
CANT7	*Tolypothrix distorta*	Gádor quarry (Almería, Spain)	Filamentous, with heterocysts and false branching

The cyanobacteria strains were preserved in both plate cultures and liquid cultures, where they were maintained in the exponential growth phase (~ 1 g L^-1^), To obtain liquid cultures, individual trichomes were transferred into sterilized Erlenmeyer flasks (0.25 L) containing BG11 media (for non-heterocystous strains) and BG110 media (for heterocystous strains) (Stanier et al., 1971). The flasks were aerated continuously with sterilized air filtered through a 0.22 µm Millex EMD Millipore™ filter, and the cultures were maintained in a room at a temperature of 25 ± 1°C under a constant irradiance of 200 µmol photons m^−2^ s^−1^. As our cyanobacterial cultures are non-axenic, the presence of a community of bacteria associated with the cyanobacterial isolates cannot be ruled out; consequently, all results related to PGP and enzymatic activities should be attributed to the combined action of the cyanobacteria and their associated bacteria. Non-axenic cultures are common for cyanobacteria, often maintained with their associated bacterial communities.

### 2.3 Evaluation of PGP properties in both cyanobacteria and heterotrophic bacteria

PGP properties and enzymatic activities were screened for both newly isolated heterotrophic bacteria and previously identified biocrust-forming cyanobacteria strains. All experiments were conducted in triplicate.

#### 2.3.1 Nitrogen-free growth

For the detection of heterotrophic bacteria capable of growth under nitrogen-free conditions, nitrogen-free medium (NFB) (Ji et al., 2014) was employed, while for cyanobacteria, the BG11_0_ medium (Rippka et al., 1979) was used. Both media were nitrogen-free, and BG11_0_ was also specifically tailored for selecting nitrogen-fixing cyanobacteria (Rippka et al., 1979). Bacteria were stricken out on the media and incubated for 7 days at 23°C. In both cases, microbial growth indicated that the strains could fix atmospheric nitrogen.

#### 2.3.2 Siderophore production

The analysis of siderophore production for both cyanobacteria and heterotrophic bacteria was done using Petri plates containing CAS (Chrome Azurol S) growth medium with agar (Schwyn and Neilands, 1987). After transferring each strain to the Petri plates, they were incubated for 3–7 days at 28°C in the absence of light. The presence of an orange halo encircling colonies served as an indicator of siderophore production.

#### 2.3.3 Phosphate and potassium solubilization activity

To detect the ability of bacteria to solubilize phosphate and potassium, respectively, the NBRIP (National Botanical Research Institute’s Phosphate) growth medium (Nautiyal, 1999) and Aleksandrow growth medium (Parmar and Sindhu, 2013) were employed. Both phosphate and potassium solubilization were observed after an incubation of 5–6 days at 28°C as a transparent halo surrounding the bacterial colony. Cyanobacterial strains were then cultured in a modified NBRIP growth medium (Nautiyal, 1999) specifically designed to limit nutrients that promote heterotrophic bacterial growthby reducing carbon sources in the medium. This adjustment helps to suppress the exponential growth of heterotrophic bacteria in cyanobacterial cultures. Subsequently, the cyanobacteria plates were incubated for 2 weeks at room temperature (25 ± 1°C) under 200 µmol photons m^−2^ s^−1^ irradiance.

#### 2.3.4 IAA production

The concentration of indole-3-acetic acid (IAA) was determined by employing the Salkowski colorimetric technique (Gordon and Weber, 1951; Rahman et al., 2010). Bacteria and cyanobacteria strains were cultured respectively in TSB and BG11 growth media supplemented with 0.25% tryptophan. Then, 1 mL of the supernatant (obtained after centrifugation of the cultures at 10,000 g for 3 min) was combined with 4 mL of the Salkowski reagent (50 mL of 35% HClO_4_ and 1 mL of 0.5 M FeCl_3_). This mixture was then incubated for 20 minutes in darkness and at room temperature. Subsequently, the developed color was quantified using a spectrophotometer at a wavelength of 530 nm (Helios Zeta UVVIS, Thermo, UK). The absorbance values were compared against a standard curve generated using IAA standard concentrations.

#### 2.3.5 Biofilm formation

To assess biofilm formation, both bacteria and cyanobacteria strains were cultured respectively in TSB and BG11 growth medium. The assay was performed as previously described (Favre-Bonté et al., 2007). Briefly, cultures were inoculated into a 96-well plate containing TSB or BG11 and incubated at 28°C for 4 days. After this period, wells were washed, and the biofilms were stained with 100 μL of 0.01% crystal violet for 10 minutes. Subsequently, the wells were washed again. Once the plate was dried, the biofilm was solubilized with 100 μL of 10 mM hydrochloric acid and quantified by measuring the absorbance at 575 nm with a microplate reader ASYS UVM-340 (Montreal Biotech, Dorval, QC, Canada). Biofilm formation measurements were conducted in triplicate.

### 2.4 Evaluation of enzymatic activities

For the enzymological analyses, several standard procedures were conducted to evaluate the enzymatic activity of the isolated microorganisms. To determine the presence of protease and lipase enzymes, cyanobacteria and heterotrophic bacteria strains were inoculated on Casein Agar medium (Harley and Prescott, 2002) and Tween^®^ 80 medium (Plou et al., 1998), respectively, and then incubated at 28°C for 5 days. Positive results, indicative of enzyme activity, were identified by the appearance of a transparent halo surrounding colonies, except for lipase activity, which was indicated by the formation of a precipitate. DNase activity for both cyanobacteria and heterotrophic bacteria was determined by culturing them in Petri plates with DNA Agar and incubating them for 5 days at 28°C. The bacteria’s ability to hydrolyse DNA was evidenced by the emergence of a transparent halo after the addition of a solution of 1M HCl, whose diameter was recorded as a semi-quantitative assessment of DNase production (Flores-Duarte et al., 2022). The protocol for measuring lipase, protease, and DNase activity in cyanobacterial strains was adapted, with the strains cultured respectively in modified Tween 80 medium, Casein Agar medium, and DNA Agar, all designed to limit nutrients for heterotrophic bacteria and reduce their exponential growth in cyanobacterial cultures (Harley and Prescott, 2002; Plou et al., 1998; Flores-Duarte et al., 2022). Starch agar plates (Scharlab) were used to assess amylase activity for both heterotrophic bacteria and cyanobacteria (Flores-Duarte et al., 2022). Lugol’s solution was added for revelation, and a transparent halo around bacterial biomass indicated amylase activity. Finally, the presence of the cytochrome-C-oxidase enzyme was determined by rubbing both cyanobacteria and heterotrophic bacteria colonies onto an Oxidase Test Disc (PanReac). The color changes to dark purple within 5 to 10 seconds indicates positive oxidase activity. A drop of 3% H_2_O_2_ was applied directly to microorganisms’ biomass to determine the presence of catalase activity (Reiner, 2010). Immediate bubble formation indicates positive catalase activity.

### 2.5 Content of total and released extracellular polymeric substances in the cyanobacterial cultures

For the determination of total EPS, 1 mL of the culture of the cyanobacterial strains (biomass with its culture medium) was taken and its carbohydrate content was quantified using the phenol-sulfuric acid assay (Dubois et al., 1956). To determine the released EPS (RPS), 5 mL of culture was centrifuged at 4,000 x g for 30 minutes, and the supernatant containing the RPS was carefully recovered. After this, the phenol-sulfuric acid assay was applied on 1 mL aliquot of this extract to quantify carbohydrate content. When the EPS amount was notably high, samples were diluted at ratios of 1:5 or 1:10 before analysis. The analysis was conducted in triplicate on day 0 and day 14 of the culture.

### 2.6 Selection and identification of bacterial isolates with the highest number of PGP properties

The heterotrophic bacterial strains exhibiting the highest number of PGP traits were selected and identified through 16S rRNA gene sequencing. A heatmap was constructed in R (version 4.2.1) by standardizing all PGP trait measurements and expressing them as percentages of the maximum value observed for each trait, to normalize different scales. This approach allowed us to compare and visualize relative differences across strains comprehensively. The variables used included quantitative data (e.g., biofilm formation), semi-quantitative data (e.g., DNase), and qualitative data with three categories (absence of the property, presence of the property, high presence of the property). The different measurement scales were converted into percentages to ensure comparability between the variables, allowing for an integrated visualization of the relative differences between samples. When strains had an equal number of PGP traits, those with different morphologies and origins were chosen to increase diversity. The bacterial genomic DNA of the selected microorganisms was extracted and purified using the PCR GenElute™ Extraction kit (Sigma) according to the manufacturer’s instructions. The following primers were used for the PCR amplification of the 16S rRNA gene containing the variable regions (V1-V9): 27F (5’-AGAGTTTGATCCTGGCTCAG-3’) (Wilmotte et al., 1993) and 1492R (5′-GGTTACCTTGTTACGACTT-3′) (Stackebrandt et al., 1993). After DNA Sanger sequencing, the raw sequences were assessed for quality using Chromas Lite v2.6.6 (Technelysium Pty Ltd). The cleaned sequences were aligned using MEGA X (Molecular Evolutionary Genetics Analysis, version 10.2.6) software, applying ClustalW for multiple sequence alignment with default parameters. The aligned sequences were analyzed using BLASTn to identify nucleotide sequence identity with the NCBI GenBank database (http://www.ncbi.nlm.nih.gov). The combination of highest identity, total score and query coverage values were used to attribute the suggested species. The final gene sequences were then submitted to GenBank database under the accession numbers MN826564.1, OR946095.1, CP140981.1 and, CP139444.1.

### 2.7 *Triticum aestivum* seed biopriming experiments

#### 2.7.1 Seed treatment

*Triticum aestivum* seeds were obtained from Cantueso Natural Seeds (Córdoba, Spain) and surface disinfected before experimentation. Initially, 1 mL of 70% ethanol was added to the seeds, which were then vortexed for 2 minutes. The mixture was centrifuged at 10,000 g for 5 minutes at 20°C, and the ethanol was subsequently discarded. Seeds were then treated with 5% bleach and placed on an orbital shaker at 150 rpm for 10 minutes. After this step, the supernatant was removed, and the seeds underwent five consecutive washes with distilled deionized water (ddH_2_O). Each wash involved vortexing, centrifugation for 5 minutes, and careful removal of the water to ensure complete elimination of any remaining disinfection agents.

Seeds were resuspended in three different biopriming inoculants and placed on a shaker for 24 hours. The microbial strains used were selected based on their previously assessed PGP properties, with priority given to those demonstrating the highest potential to enhance plant growth. The first inoculant contained *N. commune* CANT2 at a concentration of 1 g dry weight L^-1^, while the second inoculant consisted of *P. frigoritolerans* 1E at a concentration of 10^10^ CFU mL^-1^. The third inoculant was a combination of both *N. commune* CANT2 and *P. frigoritolerans* 1E at the same respective concentrations. In all cases, the microorganisms were first centrifuged and resuspended in a physiological solution (0.9% NaCl) to remove any residual nutrients from the culture medium. As a control, seeds were incubated in the physiological solution alone, without microbial inoculants.

#### 2.7.2 Experimental setup for seed germination

Following biopriming, seeds were placed in square Petri dishes containing 0.8% agar, which had been adjusted to a pH of 5.7. The dishes were then transferred to a growth chamber set at 25°C. Each treatment was replicated across seven plates, with each plate holding eight seeds (56 seeds per treatment). Seed germination was monitored daily for a total of seven days. At the end of this period, the length of both the stem and total radicle of each germinated seedling was measured using a ruler. These measurements were then used for the calculation of the germination percentage and Seed Vigour Index (SVI).

Germination percentage (Equation 1) was calculated as the proportion of seeds germinated expressed as a percentage of the total seeds tested, while Seed Vigour Indices (SVI I and SVI II) (Equations 2-3) were calculated using the formula proposed by Abdual-Baki and Anderson (1973), with modifications. For these calculations, the following formulas were employed:


(1) $$\text{Germination }\%=(\frac{Number\;of\;seeds\text{ germinated}}{Total\;number\;of\;seeds})\times 100$$

(2) SVI I=(Shoot length+Root length)×Germination %

(3) SVI II=Fresh Weight ×Germination %

### 2.8 Statistical analyses

One-way ANOVA was employed to analyze differences in biofilm production and DNase activity among heterotrophic bacterial strains, EPS production among cyanobacteria strains, and SVI I and SVI II for the germination tests. The Tukey *post-hoc* test was conducted for multiple comparisons when the results were statistically significant. All variables were previously tested for normality and homogeneity of variance using the Shapiro–Wilk and Levene’s test. In addition, a repeated-measures ANOVA was performed to assess the effect of treatments on germination percentage over time. All statistical analyses were performed using R Statistical Software (v4.2.1; R Core Team, 2021), using the *nlme* package (version 3.1-159) for repeated measures ANOVA and the *stats* package (version 4.2.1) for Tukey’s *post-hoc* test. Results were considered significant when p-value < 0.05.

## 3 Results

### 3.1 Isolation of heterotrophic bacteria from biocrust

We isolated a total of eighty-seven bacterial strains from the different biocrust types: sixteen from cyanobacteria-dominated biocrust (CB); eighteen from lichen-dominated biocrust with *Diploschistes* sp. as the dominant genus (LB); twenty-four from moss-dominated biocrust (MB); and twenty-nine from Hepatophyta-dominated biocrust (HB). Out of the eighty-seven isolated heterotrophic bacteria, twenty-six strains were selected for PGP tests, considering the diversity of their colony morphology and the various types of biocrust origins (**Supplementary Table S1**).

### 3.2 PGP properties for heterotrophic bacteria

**Figure 2:**
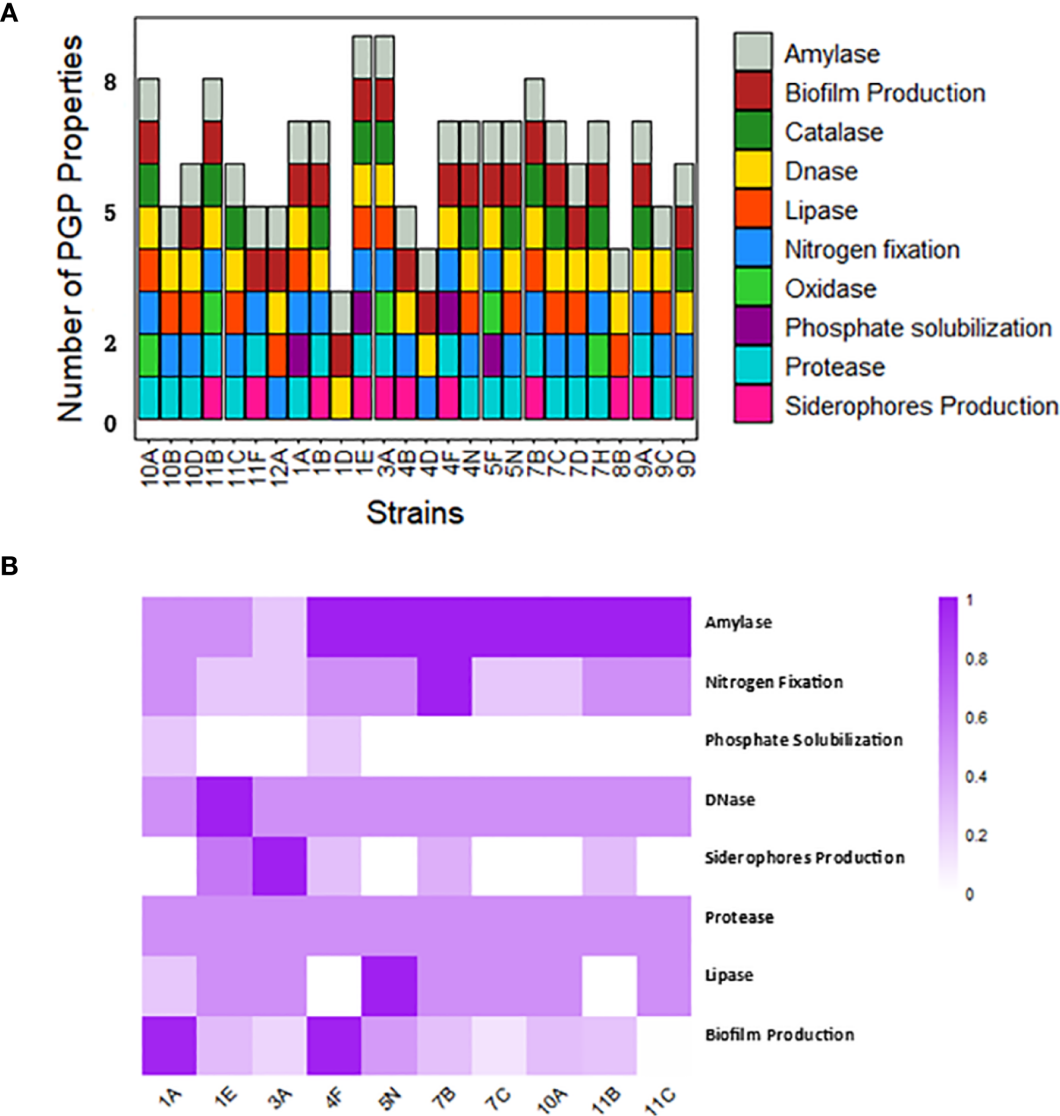
Characterization of heterotrophic bacteria. **(A)** Number of PGP properties in each bacterial strain **(B)** Heatmap of the 10 strains with the highest PGP properties, based on standardized measurements expressed as percentages.

Based on the PGP assays, each isolate showed between three and nine PGP traits (**Figure 2A**). Among the twenty-six tested isolates, eleven strains (42.3%) could produce siderophores, while only four strains (1A, 1E, 4F, and 5F) could solubilize phosphate (15.4%). Almost all strains (92.3%) demonstrated the ability to growth in nitrogen-free media, except strains 1D and 8B, and twenty-two isolates (84.6%) could produce biofilms. However, none of the isolates could produce auxin or solubilize potassium. Regarding the enzymatic activity of the tested strains, the DNase test was positive for twenty-five out of twenty-six isolates (excepting 11F), while the protease enzyme was detected in only two strains (7.7%). The lipase test was positive for fifteen isolates (57.7%), the catalase test was positive for thirteen isolates (50%), and the oxidase test for five isolates (19.2%). Finally, the amylase enzyme was found in all twenty-six strains. The heterotrophic bacterial strains with the highest number of PGP properties were 1E and 3A, each with nine properties, followed by strains 7B, 10A, and 11B, each with eight PGP properties. The strains with the fewest PGP properties were 4D and 8B, each with four properties, and 1D with only three PGP properties. In **Figure 2B**, the ten strains with the highest PGP properties are displayed, based on standardized measurements expressed as percentages. There was considerable variability among strains for certain properties (e.g., biofilm formation), while others showed less variability (e.g., protease activity).

**Figure 3:**
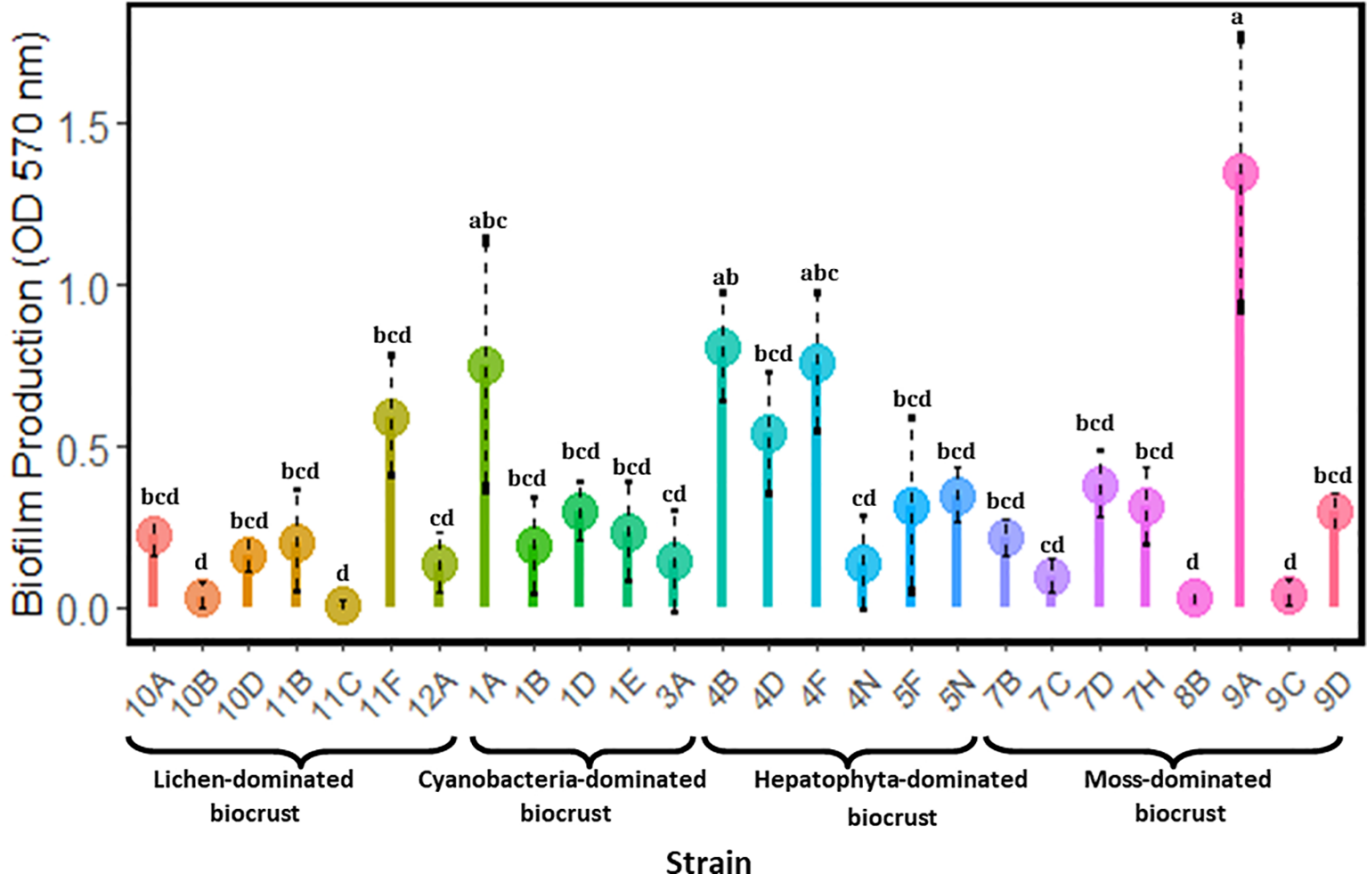
Biofilm production of heterotrophic bacteria. Different letters indicate significant differences between strains (p-value < 0.05).

The analyzed strains exhibited significant differences in biofilm production (**Figure 3**). Strain 9A consistently showed higher biofilm production compared to several other strains, highlighting its potential as a robust biofilm producer. Additionally, strains 4B and 4F exhibited significantly higher biofilm production compared to strains such as 10B and 11C. In contrast, the remaining strains did not show statistically significant differences, suggesting that their biofilm production capabilities were relatively similar.

**Figure 4:**
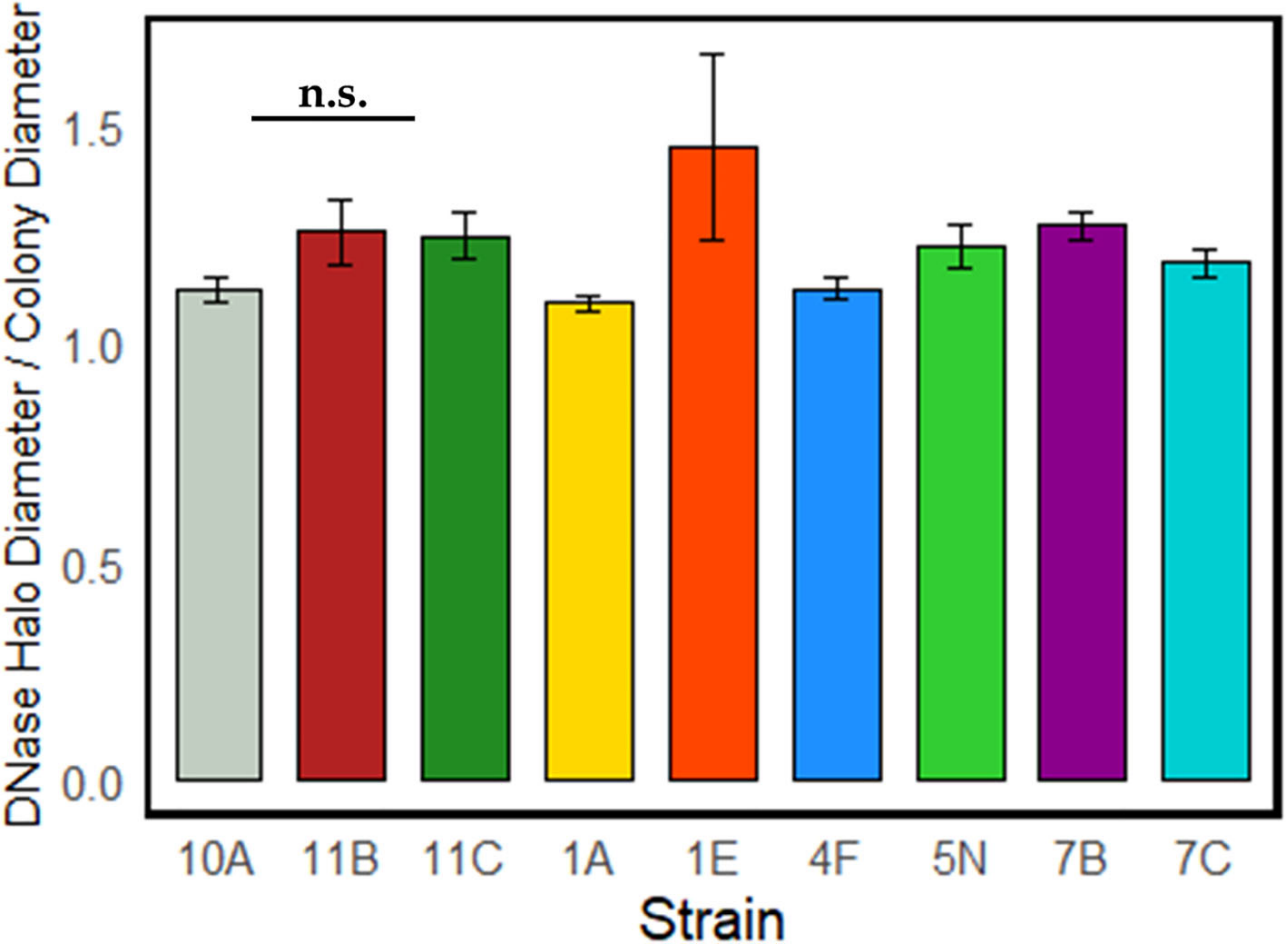
DNase activity of the strains with higher PGP properties. No statistically significant differences emerged among the strains.

**Figure 4** illustrates the DNase activity of the strains identified as the top-performing among heterotrophic bacteria, showing the ratio between the test’s positive halo and the size of the colony’s halo. This ratio averaged between 1 and 1.5 for all strains, and no significant differences were found among strains.

### 3.3 Selection of PGP heterotrophic bacteria

Among the twenty-six strains subjected to presence/absence evaluation for PGP traits, we selected ten strains based on the highest number of PGP properties. Criteria for selection involved excluding strains with similar morphology and originating from the same type of biocrust. The selected strains were: 1A, 1E, 3A, 4F, 5N, 7B, 7C, 10A, 11B, and 11C. For these strains, quantitative values are reported in **Figure 2B**. Strain 7B demonstrated the highest growth rates in nitrogen-free media, followed by 1A, 4F, 5N, 11B, and 11C. For siderophore production, strain 3A stood out, with 1E, 7B, 4F, and 11B showing progressively lower capabilities. Strains 1A and 4F exhibited similar phosphate solubilization abilities. In terms of biofilm formation, 1A and 4F were the most effective, followed by 5N, 1E, 7B, 10A, and 11B. Protease activity was uniform across all strains. Lipase activity was highest in strain 5N, lower in several others, and absent in 4F and 11B. All strains showed amylase and DNase activity, with higher amylase levels observed in several strains, and DNase activity particularly prominent in strain 1E. Although strain 3A exhibited a high number of PGP properties and notable siderophore production, it was excluded from subsequent analyses due to the lack of reliable taxonomic identification.

### 3.4 Identification of PGP heterotrophic bacteria

Identification of the heterotrophic bacteria species made on the phylogenetic analysis of the 16S rRNA gene partial sequence for the selected strains is shown in **Table 2**. Strains 1A and 4F were identified with an identity of 100% as *Bacillus atrophaeus* and strains 5N, 7B, 7C, 11B, and 11C were identified as *Bacillus anthracis* Cohn. Finally, strain 1E was identified as *Peribacillus frigoritolerans* (Delaporte and Sasson, 1967). Strains identified as *B. anthracis* were eliminated from the collection since they belong to Risk Group 3.

**Table 2:** Identification of heterotrophic bacteria selected for their PGP properties.

Strain	Species	GenBank accession number
1A	*Bacillus atrophaeus*	MN826564.1
1E	*Peribacillus frigoritolerans*	OR946095.1
3A	Not identified*	–
4F	*Bacillus atrophaeus*	CP140981.1
5N	*Bacillus anthracis*	CP139444.1
7B	*Bacillus anthracis*	CP139444.1
7C	*Bacillus anthracis*	CP139444.1
10A	Not identified*	–
11B	*Bacillus anthracis*	CP139444.1
11C	*Bacillus anthracis*	CP139444.1

All strains analyzed showed over 99.5% identity.

*Not identified due to bad sequence quality.

### 3.5 Plant growth promotion properties for cyanobacteria

**Figure 5:**
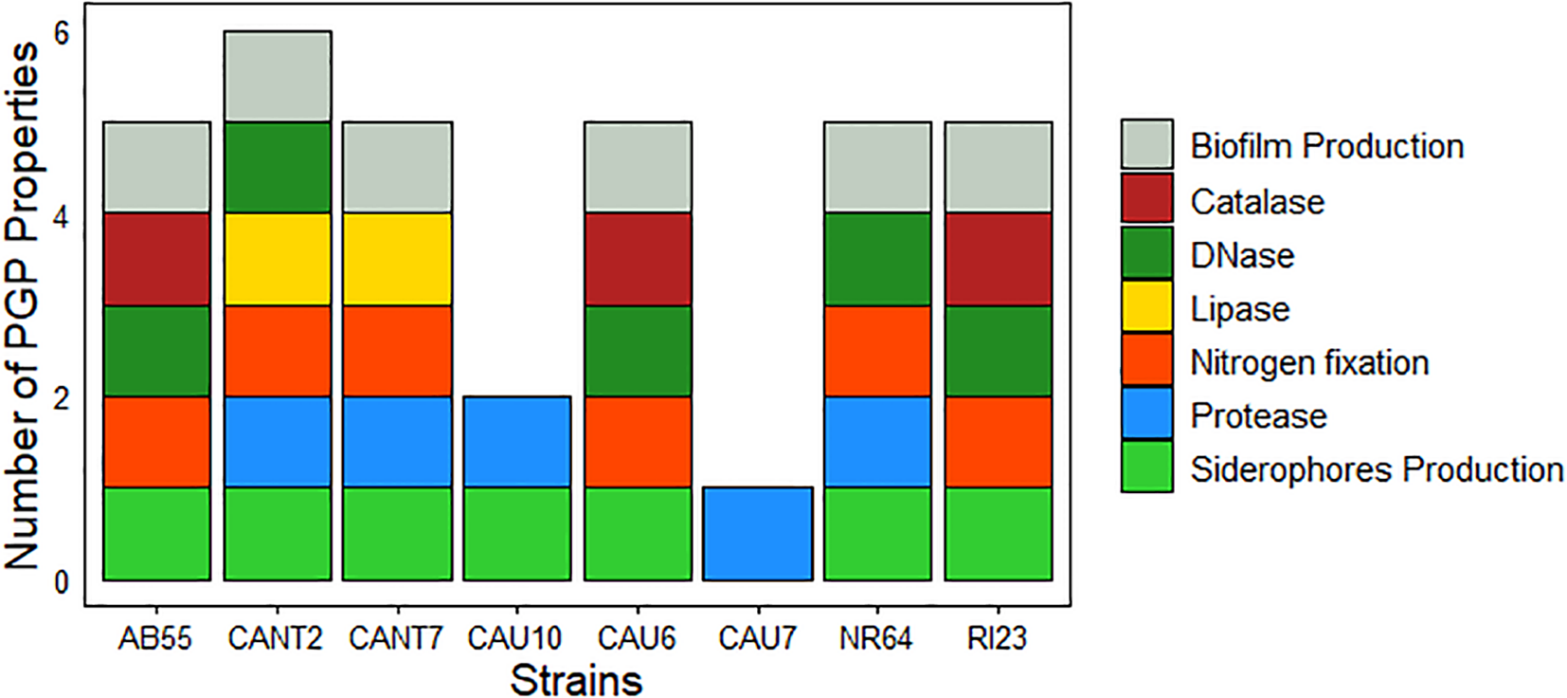
Characterization of cyanobacterial strains. Number of PGP properties for strains belonging to the species N. commune (strains AB55, CANT2, NR64, and RI23), *T. distorta* (CANT7), *S. frigidus* (CAU10), *T. desertorum* (strain CAU7), and *S. hyalinum* (CAU6).

Analysis of the PGP properties on the selected cyanobacteria showed that the strain *Nostoc commune* CANT2 had the highest number (six) of PGP traits (**Figure 5**). It is followed by *N. commune* AB55, *T. distorta* CANT7, *T.* cf. *desertorum* CAU7, *N. commune* NR64, and *N. commune* RI23, each one having five PGP traits. All tested cyanobacteria, except for *S. frigidus* CAU10 and *S. hyalinum* CAU6, were able to produce biofilms. Additionally, all strains except for *S. hyalinum* CAU6 could produce siderophores. The results were negative for both phosphate solubilization and potassium solubilization capacities considering all cyanobacteria strains tested. Regarding the enzymatic activity of the tested strains, the DNase test was positive for five strains (*N. commune* AB55, *N.* commune CANT2, *T. desertorum* CAU7, *N. commune* NR64, and *N. commune* RI23). The protease enzyme was detected in *N. commune* CANT2, *T. distorta* CANT7, *S. frigidus* CAU10, *S. hyalinum* CAU6, and *N. commune* NR64. The lipase test was positive for two strains (*N.* commune CANT2 and *T. distorta* CANT7), and the catalase test was positive for three strains (*N. commune* AB55, *T.* cf*. desertorum* CAU7, and *N. commune* RI23). No results were obtained for the amylase tests for none of the cyanobacteria due to contamination by heterotrophic bacteria on the plates.

### 3.6 EPS production by cyanobacterial strains

**Figure 6:**
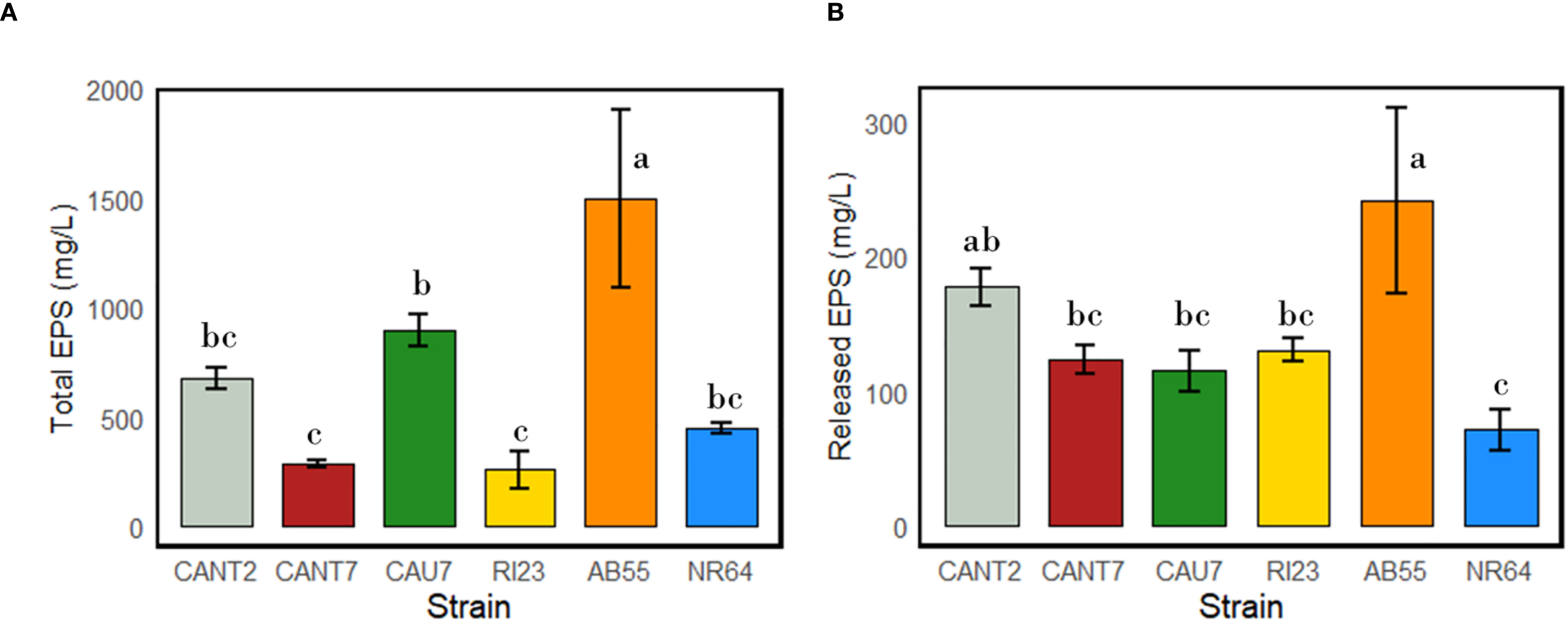
Total **(A)** and released **(B)** EPS production by cyanobacterial strains belonging to the species *N. commune* (strains AB55, CANT2, NR64, and RI23), *T. distorta* (CANT7), and *T. desertorum* (strain CAU7). Different letters indicate significant differences between strains (p-value < 0.05).

The results of the analyses for both total and released EPS (**Figure 6**) revealed substantial differences in EPS production among the cyanospheres of the strains *N. commune* CANT2, *T. distorta* CANT7, *T. desertorum* CAU7, *N. commune* RI23, *N. commune* AB55, and *N*. *commune* NR64. The strains *S. frigidus* CAU10 and *S. hyalinum* CAU6 were excluded from the EPS analyses due to their minimal PGP properties and lack of biofilm production among the cyanobacterial strains. The strain *N. commune* AB55, isolated from southern Sardinia, exhibited the highest production of both total and released EPS among all the cyanobacterial strains (1498.7 mg L^-^¹ for total EPS and 241.8 mg L^-^¹ for released EPS). Among the strains isolated from the province of Almería, *T. desertorum* CAU7 showed the highest production of total EPS (898.3 mg L^-^¹), followed by *N. commune* CANT2 (675.8 mg L^-^¹). However, *N. commune* CANT2 exhibited the highest amount of released EPS, reaching 177.1 mg L^-^¹. The strains *T. distorta* CANT7, *N. commune* RI23, and *N. commune* NR64 showed the lowest quantities of both total and released EPS. Noteworthy is that *T. desertorum* CAU 7, despite exhibiting high total EPS content, showed among the lowest quantities of released EPS.

### 3.7 Effects of biopriming on seed germination and seedling vigor index of *T. aestivum*

**Figure 7:**
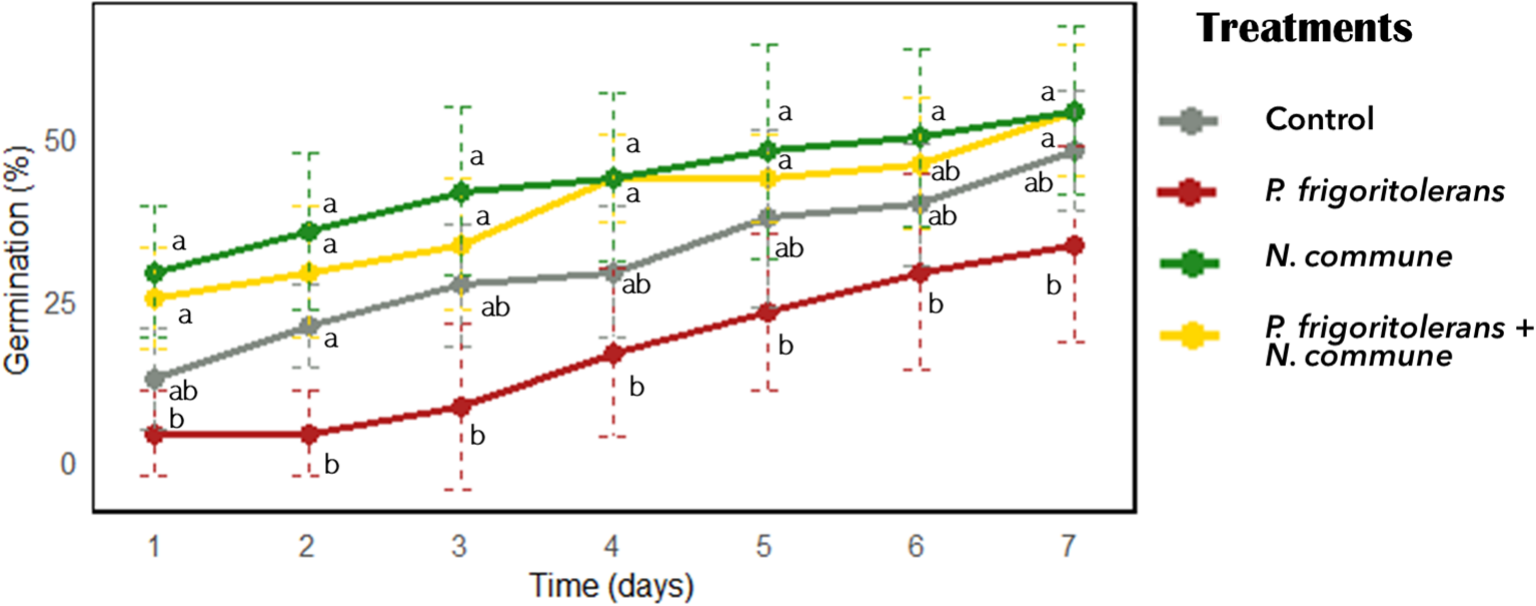
Effect of biopriming treatments on germination percentage (%) over time.

The analysis of the germination percentage in the *T. aestivum* treated seeds revealed a significant reduction in germination in the seeds bioprimed with *P. frigoritolerans* 1E compared to the control (**Figure 7**). In contrast, biopriming with the cyanobacterium *N. commune* CANT2 and the co-inoculation of both *N. commune* CANT2 and *P. frigoritolerans* did not show significant differences compared to the control. After 7 days, the germination percentage was 51.9% in the control treatment, 37.5% in the *P. frigoritolerans* biopriming treatment and reached 60.7% for seeds bioprimed with *N. commune* CANT2. Co-inoculation with *P. frigoritolerans* 1E and *N. commune* CANT2 resulted in a germination percentage of 54.7%. A repeated-measures ANOVA was conducted to evaluate the effect of treatments on germination over time. The analysis revealed a significant interaction between treatment and time (*F* = 3.012, *p* < 0.001), suggesting that the effect of time on germination varied across treatments. A significant effect of treatment was observed (*F* = 51.167, *p* < 0.0001),a and the effect of time was also significant (*F* = 15.696, *p* < 0.0001), supporting that germination changed significantly over time. However, *post hoc* analyses did not show significant differences over time for each treatment (**Figure 7**). In treatments with *N. commune* CANT2 and with both *P. frigoritolerans* and *N. commune* CANT2, a high proportion of seeds germinated from day one, resulting in a flatter temporal slope. In contrast, germination in the control and bacterial-only treatments began later, leading to a steeper increase over time.

**Figure 8:**
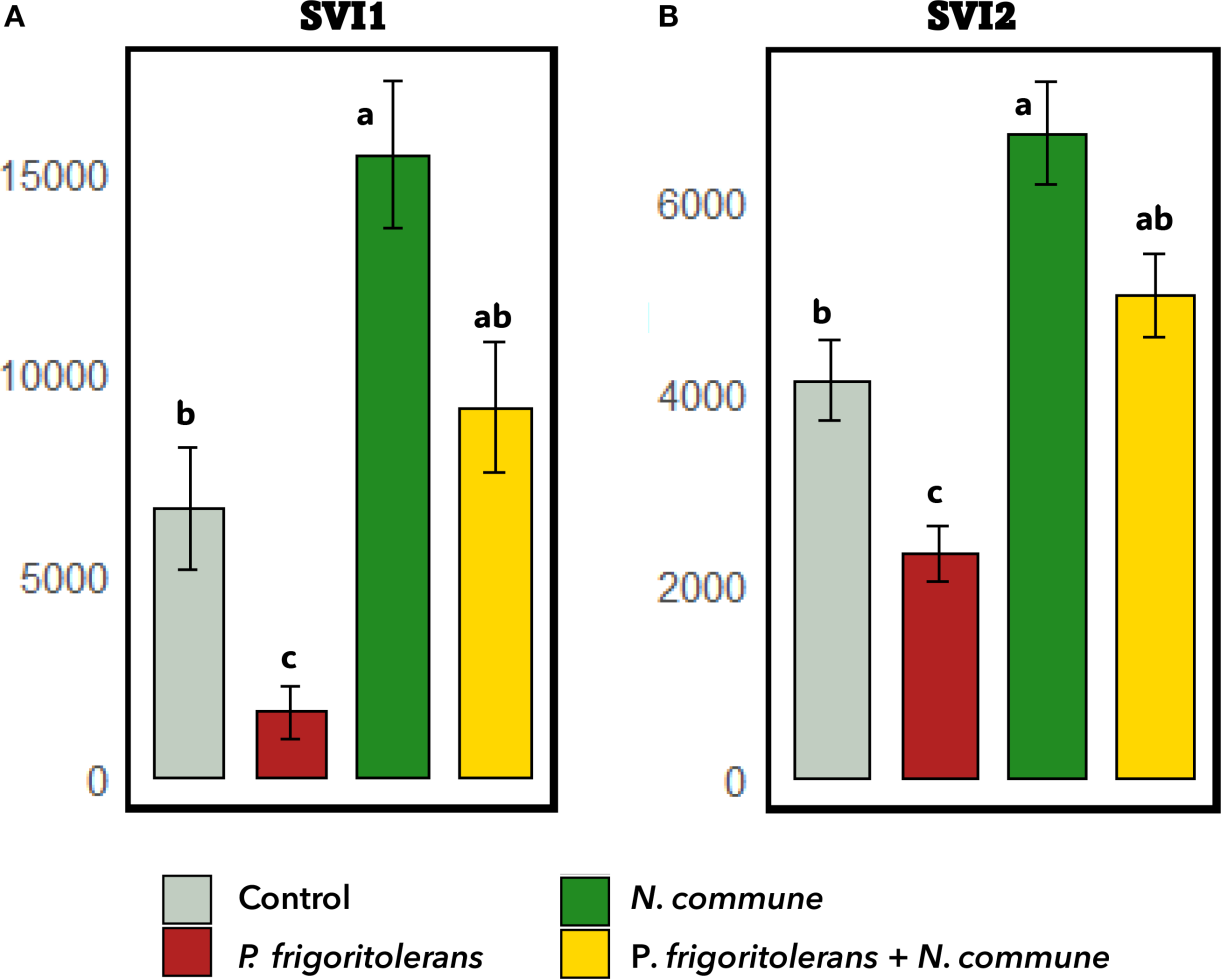
Effect of biopriming treatments on seedling vigor indices (SVI). **(A)** SVI I and **(B)** SVI II. Higher values indicate greater vigor under the respective treatments.

**Figure 8** shows that seeds subjected to *N. commune* CANT2 biopriming exhibited a significant increase in both SVI I (p < 0.001) and SVI II (*p* < 0.001) compared to the control. Conversely, biopriming with *P. frigoritolerans* 1E significantly reduced both SVI I (p = 0.023) and SVI II (p = 0.033). Nevertheless, no statistically significant differences were observed between the *N. commune* CANT2 treatment and the combined *N. commune* CANT2 and *P. frigoritolerans* 1E treatment for either SVI I (p = 0.184) or SVI II (p = 0.061).

## 4 Discussion

### 4.1 PGP characteristics and applied potential of biocrust microorganisms

In this study, heterotrophic bacteria and cyanobacteria strains isolated from biocrusts revealed several PGP traits, including siderophore production, phosphate solubilization, biofilm formation, and different enzyme activities, such as DNase, lipase, protease, oxidase and amylase. These key PGP properties have been extensively reported over years in studies on rhizosphere bacteria (Mohan and Kumar, 2019; Khan et al., 2021; Lacava et al., 2022), but similar investigations on biocrust heterotrophic bacteria are largely absent from the literature. A few studies have detailed PGP traits for cyanobacteria, particularly for species belonging to the genera *Anabaena, Arthrospira, Calothrix, Nostoc, Oscillatoria, Phormidium*, and *Tolypothrix* (Múnera-Porras et al., 2020; Toribio et al., 2020; Arahou et al., 2023; Elsheikh and Eltanahy, 2024). However, these studies focused exclusively on aquatic or rhizospheric cyanobacteria, whereas research on cyanobacteria from biocrusts remains unexplored.

Among the twenty-six heterotrophic bacteria strains evaluated for PGP properties, ten were selected based on their high number of PGP characteristics, morphological diversity, and distinct biocrust origins (**Figure 2B**). These selected strains include *B. atrophaeus* (strains 1A and 4F), *B. anthracis* (strains 5N, 7B, 7C, 11B, and 11C), and *P. frigoritolerans* (strain 1E), with notable capabilities in siderophore production, phosphate solubilization, biofilm formation, and enzymatic activities. All these genera are commonly found in dryland soils, where they play crucial roles in nutrient cycling and soil health (Ayangbenro et al., 2018; Gil et al., 2023). Additionally, some strains belonging to *Bacillus* sp. have been previously isolated from biocrusts, highlighting their ecological significance in these unique environments (Karaoz et al., 2018; You et al., 2021). However, it is important to note that the apparent dominance of *Bacillus* and *Peribacillus* in our results may be influenced by the culture-dependent approach employed, which can favor the growth of certain taxa over others present in the biocrust community. This methodological bias should be considered when interpreting these findings. The species *B. atrophaeus* is a Gram-positive, aerobic, spore-forming bacterium from the *Bacillus* genus, closely related to *Bacillus subtilis* (Nakamura, 1989). The strains belonging to this species demonstrate remarkable resistance to extreme environmental conditions due to their ability to produce highly durable spore structures that ensure their survival in harsh environments (Feshangsaz et al., 2020). *B. atrophaeus* has numerous uses in biotechnology and has been extensively studied for its applications as a biocontrol agent, in the production of industrial enzymes, as a model organism in microbiological research, and in the synthesis of bioactive compounds (Sella et al., 2015). Numerous studies report Plant Growth-Promoting Rhizobacteria (PGPR) properties for strains of *B. atrophaeus* in crops which can enhance the growth of crops such as *Chenopodium quinoa* Willd (Mahdi et al., 2021), *Zea mays* L., and *Solanum lycopersicum* L (Huang et al., 2015; Shah et al., 2020). Furthermore, inoculating crops with *B. atrophaeus* has been shown to significantly reduce the harmful impact of salt stress, enhancing overall crop resilience and growth (Mahdi et al., 2021; Hou et al., 2022). On other side, *P. frigoritolerans* is a rod-shaped Gram-positive bacterium classified initially as *Brevibacterium frigoritolerans*, belonging to the family Bacillaceae (Montecillo and Bae, 2022). Despite being less extensively studied than *B. atrophaeus*, recent literature confirms the PGP capabilities of *P. frigoritolerans.* One of the reasons for this gap in the literature is that the *Peribacillus* genus was separated from the *Bacillus* one very recently (Patel and Gupta, 2020). According to Świątczak et al. (2024), this microorganism exhibits significant PGP properties, including the production of IAA (not observed in our strain), phosphorus solubilization, ACC deaminase activity, and siderophore production. Additionally, Marik et al. (2024) reported that a strain of *P. frigoritolerans* significantly enhanced root and shoot growth in *Arabidopsis thaliana* (L.) Heynh under induced drought stress. *B. anthracis* is a Gram-positive bacterium known for its ability to form resilient spores and as the causative agent of the serious infectious disease anthrax (Turnbull et al., 2002). Although the PGP properties of *B. anthracis* strains have been well-documented and confirmed by other studies in the literature (Ali et al., 2021; Azeem et al., 2023), their application in agronomic or biotechnological experiments is severely constrained due to their potentially high pathogenicity. Consequently, we decided to exclude these strains from our collection for future field applications.

The results of the PGP properties in the tested cyanobacterial strains highlight a broad diversity in the biochemical capabilities of these microorganisms. Strain *N. commune* CANT2 stands out significantly, having six PGP properties which includes the ability to form biofilms, produce siderophores, and exhibit various enzymatic activities (DNase, protease and lipase) (**Figure 5**). Furthermore, this strain showed the second-highest concentration of released EPS (177.14 mg L^-1^) placing it just after *N. commune* AB55 (241.78 mg L^-1^). Both strains AB55 and CANT2 are characterized for their production of exopolysaccharides (Alameda-Martín et al., 2024; Pagli et al, 2024), which contribute to their biofilm-forming capacity and overall PGP potential. This suggests that CANT2 might have potential as a biofertilizer or biocontrol agent in agricultural applications, given its potential ability to support plant growth and improve nutrient availability (Saha et al., 2020; Águila-Carricondo et al., 2024). The strains *N. commune* AB55, *T. distorta* CANT7, *T. desertorum* CAU7, *N. commune* NR64, and *N. commune* RI23 follow with five PGP properties each, indicating they also have a considerable potential in promoting plant growth and serving as biostimulants. However, they differ from *N. commune* CANT2 in that *N. commune* AB55, CAU7, and RI23 lack protease activity, *T. distorta* CANT7 lacks DNase activity, and *N. commune* NR64 lacks lipase activity. Despite belonging to same species, *N. commune* strains exhibit distinct biochemical profiles that may influence their effectiveness as biofertilizers. It is important to consider how these differences may impact their performance in various environmental conditions. Most selected cyanobacteria strains shared the ability to form biofilms (except *S. frigidus* CAU10 and *T.* cf. *desertorum* CAU7) and produce siderophores (except *S. hyalinum* CAU6) which supports root colonization and iron sequestration, crucial for pathogen competition and plant health promotion (Nishanth et al., 2021; Paul et al., 2024). These findings align with previous research demonstrating the efficacy of *N. commune* in promoting plant growth and enhancing soil fertility (Román et al., 2018; Rai et al., 2019; Hata et al., 2022; Alameda-Martín et al., 2024). Bharti et al. (2017) reported that *N. commune* exhibits strong biofilm-forming capabilities. Additionally, Fresenborg et al. (2020) confirmed the production of siderophores by cyanobacteria of the genus *Nostoc.* Biocrusts usually colonize extreme environments where microorganisms must adapt to adverse conditions such as limited nutrient availability, UV radiation exposure, and fluctuations in temperature and humidity (Rodriguez-Caballero et al., 2018; Mackelprang et al., 2022). These adaptations may have endowed the microorganisms with unique properties, such as the ability to colonize challenging environments, improve nutrient availability, and protect themselves against abiotic and biotic stresses (Mackelprang et al., 2022). Future studies should include quantitative assays and adopt standardized units to enable better comparison of PGP properties across different strains and studies, while addressing the limitations of non-axenic cultures and the need for further work to clarify the specific roles of enzymatic activities in PGPM function.

### 4.2 Effects of biopriming with biocrust microorganisms on seed germination and seedling vigor

Despite the PGP properties of *P. frigoritolerans* 1E, the results of the germination experiment conducted on *T. aestivum* showed that the biopriming treatment with *P. frigoritolerans* 1E significantly reduced germination percentage and vigor indices compared to the control. This suggests a potential negative impact of the bacterium on early seed development, possibly due to resource competition, the production of inhibitory compounds, or a stress response in the plant (Darrasse et al., 2010; Kuzyakov and Xu, 2013; Bai et al., 2018). Conversely, *N. commune* biopriming treatments improved both vigor indices, indicating a potential beneficial role in promoting growth. This positive effect might be linked to the production of bioactive metabolites, such as organic acids, enzymes, or plant hormones (e.g., auxins and cytokinins), which could stimulate seedling growth (Toribio et al., 2020; Nowruzi et al., 2021; Begum et al., 2022; Alameda-Martín et al., 2024). This is consistent with previous studies reporting similar effects for *Nostoc* sp. strains. In a previous study, biopriming with *N. commune* CANT2 was found to increase radicle elongation in the annual plant species *Stipa capensis* (Alameda-Martín et al., 2024). It has also been observed that foliar sprays with *N. piscinale* suspensions promote earlier leaf development in *Zea mays*, with a single application significantly benefiting overall plant growth (Ördög et al., 2021). Similarly, inoculation of soybean plants with *N. muscorum* and *N. rivulare* has resulted in notable improvements in growth parameters such as plant height, leaf area, plant weight, and legume weight (Sholkamy et al., 2015). The seed biopriming with both *P. frigoritolerans* 1E and *N. commune* CANT2 resulted in a germination rate similar to the control, suggesting that *N. commune* CANT2 may have mitigated the negative effects of *P. frigoritolerans* 1E. This could be due to an interaction between the two microorganisms that mitigates the bacterium’s potential inhibitory effects (Gören-Sağlam, 2021; Poveda, 2021; Grabowski et al., 2024). However, the lack of significant differences between the co-inoculation and *N. commune* CANT2 alone in terms of vigor indices suggests that the positive effect of the cyanobacterium may be masking a negative effect of P*. frigoritolerans* 1E, which, when applied alone, showed even lower values than the control.

Considering both the results of the PGP properties and the biopriming application, further studies should investigate the interaction dynamics between cyanobacteria and heterotrophic bacteria to optimize their use in agriculture, evaluating strategies that maximize the PGP properties of these microorganisms and minimize any potential adverse effects on plant development. It is important to note that not all isolated strains will necessarily have a positive effect on plant growth; therefore, continuous isolation and characterization are needed to identify strains with actual beneficial potential. These combined properties endow these microorganisms with significant potential to be used as microbial inoculants to enhance plant growth in agricultural settings, contributing to sustainability and reducing the environmental impact of conventional agriculture. This approach could be particularly beneficial for agriculture in dryland environments, where the resilience of cyanobacteria to harsh conditions could help improve soil fertility and crop productivity (Chamizo et al., 2018). Although the *Peribacillus* strain used in this study did not enhance germination in the target species, it might exhibit species-specific effects and prove effective in other contexts (Alameda-Martín et al., 2024). Future studies should also test a broader set of cyanobacterial isolates, including pure cultures, to confirm the reproducibility of PGP traits, and clarify the relative importance of enzymatic activities versus nutrient mobilization. Assessing tolerance to salt, temperature, and drought could further identify strains best suited for challenging environments. Exploring synergistic combinations of cyanobacteria and associated heterotrophic bacteria may reveal ways to enhance both plant growth and stress tolerance under diverse agronomic conditions.

## 5 Conclusion

In this study, we demonstrate that biocrusts from semi-arid environments represent a promising, yet underexplored, reservoir of plant growth-promoting microorganisms. From 87 bacterial isolates, 26 strains exhibiting multiple PGP traits were identified, alongside 6 cyanobacterial strains, revealing a wide functional diversity. Notably, strains like *P. frigoritolerans* 1E, *B. atrophaeuS* 1A, and *B. atrophaeus* 4F showed a particularly broad spectrum of beneficial properties, positioning them as strong candidates for development as biofertilizers. Similarly, the cyanobacterium *N. commune* CANT2 emerged as a promising inoculant, significantly enhancing seedling vigor, in contrast to *P. frigoritolerans* 1E, which negatively affected early plant development. These findings highlight that the expression and effectiveness of PGP traits can be context-dependent and may vary between microbial taxa and plant growth stages. In contrast to many studies focusing on conventional commercial biofertilizers, our findings suggest that biocrust-derived microorganisms—both heterotrophic bacteria and cyanobacteria—offer unique functional capacities that may not be evident in early germination assays alone. Therefore, future studies should consider longer-term plant responses and explore functional comparisons with commercial strains. Overall, our work contributes to positioning biocrusts as a novel and valuable microbial niche for the development of bioinoculants, advancing sustainable agriculture while reducing dependence on chemical inputs.

## Data Availability

The original contributions presented in the study are included in the article/**Supplementary Material**. Further inquiries can be directed to the corresponding author.

## Supplementary material

Supplementary Table 1 Morphological characteristics of heterotrophic bacteria colonies isolated from different biocrust types (CB= cyanobacteria-dominated biocrust, LB= lichen-dominated biocrust with Diploschistes sp. as the dominant species, MB= moss-dominated biocrust, HB= Hepatophyta-dominated biocrust). Selected strains for PGP analysis are highlighted in bold.
