# Supplementary material for: Unlocking the potential of biocrust microorganisms in agriculture: cyanobacteria and heterotrophic bacteria with plant growth-promoting properties
Source: Front Plant Sci. 2025 Sep 24;16:1659217. doi: 10.3389/fpls.2025.1659217 (PMC12506096; doi:10.3389/fpls.2025.1659217)
Supplement: Supplementary Table 1 — Morphological characteristics of heterotrophic bacteria colonies isolated from different biocrust types (CB= cyanobacteria-dominated biocrust, LB= lichen-dominated biocrust with Diploschistes sp. as the dominant species, MB= moss-dominated biocrust, HB= Hepatophyta-dominated biocrust). Selected strains for PGP analysis are highlighted in bold. [file Table1.docx]

**Supplementary materials**

*Table S1. Morphological characteristics of heterotrophic bacteria colonies isolated from different biocrust types (CB= cyanobacteria-dominated biocrust, LB= lichen-dominated biocrust with Diploschistes* *sp. as the dominant species, MB= moss-dominated biocrust, HB= Hepatophyta-dominated biocrust). Selected strains for PGP analysis are highlighted in bold.*

| **Id** | **Shape** | **Elevation** | **Margin** | **Colour** | **Size** | **Opacity** | **Texture** | **Surface** | **Type Biocrust** | **Medium Concentration** |
| --- | --- | --- | --- | --- | --- | --- | --- | --- | --- | --- |
| **1A** | **irregular** | **crateriform** | **entire** | **white** | **5 mm** | **opaque** | **slimy** | **smooth** | **CB** | **1x** |
| **1B** | **circular** | **convex** | **entire** | **milky** | **3-4 mm** | **opaque** | **buttery** | **rough** | **CB** | **1x** |
| 1C | circular | raised | ondulate | white | 1 mm | opaque | buttery | smooth | CB | 1x |
| **1D** | **circular** | **convex** | **ondulate** | **milky** | **1 mm** | **translucent** | **buttery** | **smooth** | **CB** | **1x** |
| **1E** | **circular** | **convex** | **ondulate** | **white** | **4 - 1 mm** | **opaque** | **buttery** | **rough** | **CB** | **1x** |
| 1F | circular | raised | ondulate | white | 1 mm | opaque | buttery | smooth | CB | 1x |
| 1G | circular | raised | curled | white | 0.5 - 1 mm | transparent | buttery | smooth | CB | 1x |
| 2A | circular | convex | ondulate | milky | 2 - 3 mm | opaque | buttery | rough | CB | 0.1x |
| 2B | irregular | umbonate | ondulate | milky | 3 - 5 mm | opaque | buttery | rough | CB | 0.1x |
| 2C | circular | raised | ondulate | milky | 1- 4 mm | opaque | buttery | smooth | CB | 0.1x |
| 2D | circular | convex | entire | milky | 1 - 2 mm | opaque | buttery | smooth | CB | 0.1x |
| 2E | circular | raised | entire | milky | 0.5 1 mm | translucent | buttery | smooth | CB | 0.1x |
| 2F | irregular | convex | entire | orange | 0.5 mm | translucent | buttery | smooth | CB | 0.1x |
| **3A** | **irregular** | **raised** | **ondulate** | **milky** | **1-4 mm** | **opaque** | **buttery** | **smooth** | **CB** | **0.01x** |
| 3B | irregular | flat | ondulate | white | 1-3 mm | transparent | buttery | smooth | CB | 0.01x |
| 3C | circular | convex | ondulate | milky | 0.5-1 mm | translucent | buttery | smooth | CB | 0.01x |
| 4A | irregular | crateriform | entire | white | 2- 3 mm | opaque | slimy | smooth | HB | 1x |
| **4B** | **circular** | **convex** | **entire** | **milky** | **4 mm** | **opaque** | **buttery** | **rough** | **HB** | **1x** |
| 4C | circular | raised | ondulate | milky | 1 - 3 mm | opaque | buttery | rough | HB | 1x |
| **4D** | **circular** | **convex** | **entire** | **milky** | **0.5 mm** | **translucent** | **buttery** | **smooth** | **HB** | **1x** |
| 4E | circular | raised | entire | milky | 1 mm | translucent | buttery | smooth | HB | 1x |
| **4F** | **irregular** | **raised** | **ondulate** | **milky** | **2 - 4 mm** | **opaque** | **buttery** | **smooth** | **HB** | **1x** |
| 4G | circular | convex | ondulate | milky | 0. 5 - 2 mm | opaque | buttery | rough | HB | 1x |
| 4H | circular | convex | entire | white | 1.2 mm | opaque | slimy | smooth | HB | 1x |
| 4I | circular | convex | ondulate | milky | 2 - 5 mm | opaque | buttery | rough | HB | 1x |
| 4L | circular | convex | entire | milky | 0.2 - 1 mm | translucent | buttery | smooth | HB | 1x |
| 4M | circular | flat | ondulate | white | 2 - 4 mm | translucent | slimy | smooth | HB | 1x |
| **4N** | **circular** | **flat** | **entire** | **cream** | **0.5 mm** | **translucent** | **buttery** | **smooth** | **HB** | **1x** |
| 4O | circular | convex | entire | yellow | 1 mm | translucent | buttery | smooth | HB | 1x |
| 5A | irregular | flat | entire | white | 0.5 - 2 mm | trasparent | buttery | smooth | HB | 0.1x |
| 5B | circular | convex | entire | milky | 0.5 - 1 mm | opaque | buttery | smooth | HB | 0.1x |
| 5C | circular | convex | ondulate | milky | 0.5 - 1 mm | translucent | buttery | smooth | HB | 0.1x |
| 5D | irregular | raised | ondulate | cream | 2-4 mm | translucent | buttery | rough | HB | 0.1x |
| 5E | circular | raised | entire | milky | 1 mm | translucent | buttery | smooth | HB | 0.1x |
| **5F** | **circular** | **raised** | **entire** | **milky** | **0.5 - 2 mm** | **translucent** | **buttery** | **smooth** | **HB** | **0.1x** |
| 5G | circular | raised | curled | white | 0.5 - 1 mm | transparent | buttery | smooth | HB | 0.1x |
| 5H | circular | convex | entire | milky | 0. 5 mm | opaque | buttery | smooth | HB | 0.1x |
| 5I | irregular | flat | ondulate | white | 2-5 mm | opaque | buttery | rough | HB | 0.1x |
| 5L | circular | convex | entire | milky | 1 mm | opaque | buttery | smooth | HB | 0.1x |
| 5M | circular | raised | entire | milky | 2 mm | opaque | buttery | rough | HB | 0.1x |
| **5N** | **circular** | **raised** | **entire** | **milky** | **0.5-1 mm** | **opaque** | **buttery** | **rough** | **HB** | **0.1x** |
| 5O | circular | convex | entire | milky | 0.5 - 1 mm | trasparent | buttery | smooth | HB | 0.1x |
| 5P | circular | convex | entire | yellow | 1 mm | iridiscent | buttery | smooth | HB | 0.1x |
| 6A | circular | convex | entire | white | 0.5 mm | translucent | buttery | smooth | HB | 0.01x |
| 6B | circular | raised | ondulate | orange | 0.5 - 1 mm | translucent | embedded | smooth | HB | 0.01x |
| 7A | irregular | raised | ondulate | white | 0.5 mm | opaque | buttery | smooth | MB | 1x |
| **7B** | **circular** | **convex** | **ondulate** | **milky** | **2mm** | **opaque** | **buttery** | **rough** | **MB** | **1x** |
| **7C** | **circular** | **flat** | **ondulate** | **white** | **1 mm** | **opaque** | **buttery** | **smooth** | **MB** | **1x** |
| **7D** | **circular** | **raised** | **curled** | **milky** | **3-5 mm** | **translucent** | **buttery** | **smooth** | **MB** | **1x** |
| 7E | irregular | flat | entire | milky | 0.5 mm | trasparent | buttery | smooth | MB | 1x |
| 7F | irregular | convex | entire | white | 0. 5 mm | translucent | buttery | smooth | MB | 1x |
| 7G | irregular | flat | ondulate | white | 0.5 mm | opaque | buttery | rough | MB | 1x |
| **7H** | **irregular** | **convex** | **entire** | **cream** | **0.5 - 1 mm** | **trasparent** | **buttery** | **smooth** | **MB** | **1x** |
| 7I | circular | flat | curled | milky | 0.5 - 1 mm | trasparent | buttery | smooth | MB | 1x |
| 8A | circular | raised | curled | white | 0.5-2 mm | transparent | buttery | smooth | MB | 0.1x |
| **8B** | **irregular** | **flat** | **entire** | **white** | **3- 4 mm** | **opaque** | **buttery** | **smooth** | **MB** | **0.1x** |
| 8C | irregular | raised | ondulate | milky | 0.5-2 mm | translucent | buttery | smooth | MB | 0.1x |
| 8D | circular | convex | entire | milky | 1 - 3 mm | opaque | buttery | rough | MB | 0.1x |
| 8E | circular | raised | ondulate | cream | 1 - 3 mm | opaque | buttery | smooth | MB | 0.1x |
| 8F | circular | flat | ondulate | white | 1 -2 mm | opaque | buttery | rough | MB | 0.1x |
| 8G | circular | convex | entire | milky | 1 - 2 mm | opaque | buttery | rough | MB | 0.1x |
| 8H | circular | raised | ondulate | orange | 0.5 - 1 mm | translucent | embedded | smooth | MB | 0.1x |
| **9A** | **circular** | **raised** | **entire** | **yellow** | **1 mm** | **opaque** | **buttery** | **rough** | **MB** | **0.01x** |
| 9B | irregular | raised | entire | pink | < 0.5 mm | translucent | buttery | smooth | MB | 0.01x |
| **9C** | **circular** | **convex** | **ondulate** | **white** | **1-2 mm** | **opaque** | **embedded** | **rugoso** | **MB** | **0.01x** |
| **9D** | **circular** | **convex** | **entire** | **white** | **0.5 mm** | **translucent** | **buttery** | **smooth** | **MB** | **0.01x** |
| 9E | circular | convex | entire | milky | < 0. 5 mm | trasparent | buttery | smooth | MB | 0.01x |
| 9F | circulare | convex | entire | yellow | < 0. 5 mm | iridiscent | buttery | smooth | MB | 0.01x |
| 9G | irregular | raised | entire | milky | 1 - 3 mm | opaque | buttery | smooth | MB | 0.01x |
| **10A** | **irregular** | **crateriform** | **entire** | **white** | **2 - 3 mm** | **opaque** | **slimy** | **smooth** | **LB** | **1x** |
| **10B** | **circular** | **raised** | **ondulate** | **milky** | **2- 3 mm** | **opaque** | **buttery** | **rough** | **LB** | **1x** |
| 10C | irregular | umbonate | ondulate | white | 3 - 5 mm | opaque | slimy | smooth | LB | 1x |
| **10D** | **irregular** | **convex** | **ondulate** | **milky** | **2 mm** | **translucent** | **buttery** | **smooth** | **LB** | **1x** |
| 10E | circular | raised | curled | milky | 0.5 mm | transparent | buttery | smooth | LB | 1x |
| 11A | irregular | flat | ondulate | white | 1 - 5 mm | opaque | buttery | rough | LB | 0.1x |
| **11B** | **irregular** | **convex** | **curled** | **milky** | **3 mm** | **opaque** | **buttery** | **smooth** | **LB** | **0.1x** |
| **11C** | **irregular** | **flat** | **ondulate** | **milky** | **3 - 5 mm** | **opaque** | **buttery** | **smooth** | **LB** | **0.1x** |
| 11D | irregular | crateriform | entire | white | 0. 5 - 2 mm | opaque | slimy | rough | LB | 0.1x |
| 11E | circular | convex | entire | white | 1 - 2 mm | translucent | buttery | smooth | LB | 0.1x |
| 11F | circular | flat | entire | yellow | 0. 5 mm | iridiscent | buttery | smooth | LB | 0.1x |
| 11G | circular | flat | entire | yellow | 0.5 mm | iridiscent | buttery | smooth | LB | 0.1x |
| 11H | circular | raised | ondulate | orange | 0.5 - 1 mm | translucent | embedded | smooth | LB | 0.1x |
| **12A** | **circular** | **convex** | **entire** | **milky** | **0.5- 2 mm** | **opaque** | **buttery** | **smooth** | **LB** | **0.01x** |
| 12B | circular | convex | ondulate | milky | 1- 4 mm | opaque | buttery | smooth | LB | 0.01x |
| 12C | circular | raised | ondulate | orange | 0.5 - 1 mm | translucent | embedded | smooth | LB | 0.01x |
| 12D | circular | raised | ondulate | orange | 0.5 - 1 mm | translucent | embedded | smooth | LB | 0.01x |
| 12E | circular | raised | ondulate | orange | 0.5 - 1 mm | translucent | embedded | smooth | LB | 0.01x |
